# Influence of Post Mortem Muscle Activity on Turkey Meat Quality

**DOI:** 10.3389/fvets.2022.822447

**Published:** 2022-02-21

**Authors:** Emily M. Leishman, Ryley J. Vanderhout, Nienke van Staaveren, Shai Barbut, Jeff Mohr, Benjamin J. Wood, Christine F. Baes

**Affiliations:** ^1^Department of Animal Biosciences, University of Guelph, Guelph, ON, Canada; ^2^Department of Food Science, University of Guelph, Guelph, ON, Canada; ^3^Hybrid Turkeys, Suite C, Kitchener, ON, Canada; ^4^School of Veterinary Science, University of Queensland, Gatton, QLD, Australia; ^5^Vetsuisse Faculty, Institute of Genetics, University of Bern, Bern, Switzerland

**Keywords:** breast meat, genetics, Meleagris gallopavo, poultry, season, slaughter, wing flapping

## Abstract

Wing flapping and body movement can occur during the slaughter of poultry. Wing movement and flapping are driven primarily by the breast muscles (*Pectoralis major and minor*), and this muscle activity may have implications for meat quality. The objective of this study was to evaluate turkey post mortem activity during slaughter at a commercial poultry processing plant. Post mortem activity (during bleeding) was scored on 5,441 male turkeys, from six different genetic lines, using a 1–4 scale from none to severe wing flapping. Meat quality was measured on these birds in terms of pH (initial, ultimate, delta or change), color (L^*^, a^*^, b^*^), and physiochemical traits (drip loss, cooking loss, shear force). Linear mixed models were used to analyze the effect of activity (score 1–4), genetic line (A–F), and season (summer vs. autumn) on the nine meat quality traits. Post mortem activity influenced a^*^, drip loss, and shear force although the magnitude of the effects was small. There was an effect (*P* < 0.05) of genetic line on all the meat quality traits except for L^*^, cooking loss, and shear force. In general, larger, faster-growing lines had higher pH, but the relationship between the lines for the other traits is not as clear. Season affected all the meat quality traits, except for pH_delta_, with meat having a higher pH, L^*^, b^*^, drip loss, cooking loss, and shear force in the summer. This study provides an exploratory assessment of post mortem activity in turkeys and identifies meat quality traits which are most affected while also accounting for the effects of genetic line and season. Although identified effect sizes are small, the cumulative effect on turkey meat quality may be more substantial.

## Introduction

During slaughter, electrical stunning is a common practice in poultry processing plants to produce an instantaneous unconsciousness before bleeding, which may or may not be reversible, as part of humane slaughter practices (1, 2). After stunning, the bird is bled by severing vessels of the neck which stops the supply of oxygen to the brain and results in death (1). Muscle activity can occur during slaughter, both pre- and post-mortem, which has consequences for the carcass and meat quality. Muscle activity, usually observed as wing flapping, can occur in both under-stunned and adequately stunned birds (3, 4). This is an issue of particular importance in turkey flocks, since variability in bird size or body weight may cause birds to be stunned unevenly. When birds are not stunned sufficiently to induce irreversible unconsciousness they may be more likely to flap or move during the slaughter process (4). Even in adequately stunned broiler chickens (leading to cardiac arrest), wing flapping has been shown to occur in 50% of birds, likely due to cardiac fibrillations during stunning (3).

This muscle activity shown during wing flapping can cause carcass damage and negatively affect meat quality, which reduces the value of the carcass (5, 6). The most common defects associated with these convulsions are hemorrhaging, blood spots, broken bones, and red wing tips (7). Since wing flapping involves use of the breast muscles, birds that flap during slaughter may experience changes in the acid/base balance and quality of their breast meat compared to immobile birds. On a physiological level, wing flapping increases plasma corticosterone, creatine kinase, lactate dehydrogenase, AMPK-activated protein kinase, acetyl-CoA carboxylase phosphorylation in broilers (8). This results in rapid glycolysis and accumulation of lactic acid, which rapidly reduces meat pH (8). Rapid drops in meat pH soon after slaughter, in combination with a warm carcass, is implicated in a number of meat quality defects. This is because pH is tightly linked to color, water-holding capacity, and other sensory properties (9, 10). In this case, meat is usually more red, lighter in color, with higher moisture loss and this has negative implications for value and consumer acceptance (8, 11, 12). However, most studies assessing muscle activity and meat quality are concerned with pre-slaughter movement.

Like pre-slaughter muscle activity, convulsions during bleeding are believed to accelerate the use of adenosine triphosphate (ATP) in the muscle and this increases the rate of pH decline (13). This effect has been shown in turkeys which were either restrained or allowed to struggle freely during exsanguination, and although struggling birds had significantly shorter rigor times, there was no apparent effect on cooking loss or shear force (13). In broilers, the impact of muscle activity during bleeding on carcass damage has been studied. While there was no discernable effect of post mortem activity on red wing tips, number of broken bones or on meat quality measures (e.g., pH, L^*^, b^*^, cook yield, shear force), breast meat redness (a^*^) was higher in decapitated birds that had higher activity (e.g., wing flapping) than conventionally killed birds with lower activity after slaughter (5).

While muscle activity (pre- or post-slaughter) may affect turkey meat quality, there are other important factors to consider, such as the effect of the environment and genetics. The effect of environmental conditions, such as ambient temperature, are well-documented on poultry meat quality (14, 15). In particular, high ambient temperatures can accelerate pH decline in the meat post mortem which typically reduces water-holding capacity and degrades meat pigment (6, 16). For this reason, several studies have found that certain meat quality attributes (e.g., color, pH, water-holding capacity) can be affected depending on the season (i.e., summer vs. winter) the birds were processed (17, 18). Given the distinct seasonal changes that occur in Canada, it is reasonable to assume that this variable should be accounted for when data collection spans many months. Aside from environmental considerations, different poultry genotypes differ in their meat quality attributes which represents another source of variation that should be accounted for in studies containing multiple genetic lines (12, 19–22). Additionally, chickens with different genotypes were shown to behave differently while shackled, which could influence their post mortem activity levels (23). For these reasons, it has been suggested for future research to investigate the influence of genotype and slaughter conditions on turkey meat quality (22).

Several older studies have considered pre-slaughter (24, 25) and post-mortem (13) activity on meat quality in turkeys. However, the prevalence of post mortem muscle activity in commercial turkey processing plants has not often been reported in modern turkey lines and it is still unclear how different levels of post mortem activity may affect meat quality. The primary objectives of this study were (a) to assess the level of post mortem muscle activity in turkeys during conventional slaughter procedures, (b) determine if varying levels of muscle activity influence meat quality traits, and (c) evaluate the effects of season and genetic line.

## Materials and Methods

All protocols complied with the guidelines of the Canadian Council on Animal Care and were approved by the University of Guelph Animal Care Committee (AUP 3782).

### Animals

Data was collected from 5,441 male turkeys from six different genetic lines (lines A–F), from June to November 2019. The turkeys were raised under identical housing and management conditions at two farms and transported (115–130 km) to a commercial poultry processing facility in Ontario, Canada. Fasting, transport, and lairage conditions were consistent across the genetic lines. Lines A and B were dam-line birds that were selected for body weight and reproductive traits. Line C was a dam line selected for reproductive traits. Line D was a small, slow-growing line. Lines E and F were sire lines selected predominately for body weight, meat yield, and feed efficiency. Birds were slaughtered between 20 and 24 weeks of age across all genetic lines.

During processing, the birds were electrically stunned and then exsanguinated. Stunning voltage was based on average liveweight for birds <9 kg (24–28 V), 9–13 kg (28–32 V), and >13 kg (32–36 V). Birds were then scalded, defeathered, and eviscerated before moving to a water chiller (2 h) and kept refrigerated for 24 h (4°C) before deboning and further meat quality measurements were collected.

### Post Mortem Activity

Videos of the birds on the slaughter line were recorded (Hero 5, GoPro, San Mateo, CA, USA). Activity was scored from these videos by the same observer using a four-category scoring system adapted from McNeal et al. (5). Activity scores (Table 1) were determined for each bird once they reached the same point on the slaughter line (post-exsanguination, immediately before scalding).

**Table 1:** Scoring scheme for post mortem carcass activity in turkeys; adapted from McNeal et al. (5).

**Score**	**Description**
1	None to minimal muscle quivering
2	Mild wing flapping
3	Moderate spasmodic body movement, curled body posture
4	Severe wing flapping and full body movement

Intra-observer reliability was calculated to ensure reliable consistent scores. The observer scored videos from five different slaughter days (~500 birds/day) in two separate sessions which were several days apart. Spearman correlations, simple and weighted kappa statistics were calculated to assess reliability of the scores between the two sessions. Kappa values were interpreted following the suggested arbitrary classifications where κ ≤ 0.20 is “poor,” 0.21 ≤ κ ≤ 0.40 is “fair,” 0.41 ≤ κ ≤ 0.60 is “moderate,” 0.61 ≤ κ ≤ 0.80 is “substantial,” and κ > 0.80 is “good” (26, 27).

### Meat Quality Measurements

Measurements were obtained during processing at the slaughter line and after chilling. Due to the nature of the study and capacity of the abattoir, meat quality measurements were only collected on a proportion of the birds that were scored for post mortem activity (*N* = 817–895 depending on the trait). Measurements included pH, color, and physiochemical characteristics which are explained in more detail in the following section. The initial pH of breast meat samples was taken at 45 min post mortem, from the cranial portion of the breast filet (pH_initial_, Portable pH meter, Hanna Instruments, Woonsocket, RI, USA). Carcasses were then water chilled (2–5°C) for ~2 h followed by storage in a commercial chiller at 4°C for 24 h before recording the other meat quality measurements.

The ultimate pH measurement was taken from the *Pectoralis major* (breast filet) at 24 hr post mortem (pH_ultimate_). The pH_ultimate_ was subtracted from the pH_initial_ to calculate the difference between the two time points (pH_delta_). Trichromatic coordinates [L^*^, a^*^, and b^*^, (28)] were measured using D50 illumination on the dorsal side of the deboned breast muscle (Nix Pro Colorimeter, Hamilton, ON, CA).

The physiochemical traits, drip loss (%), cooking loss (%), and shear force (N), were also analyzed. In brief, drip loss was determined by placing a 13 ± 1 g *Pectoralis major* sample inside a drip loss collection tube and measuring the initial and 72 h weight of the sample after refrigerated storage. For cooking loss and shear force, 200 g samples were cut from the breast muscle at 24 h post mortem and frozen at −20°C until they were defrosted and heated to an internal temperature of 72°F (determined via a thermocouple) while wrapped in aluminum foil to prevent crust formation. Cooking loss was calculated as the percent difference between the initial weight of the raw sample and final weight after cooking. To measure shear force, the cooked samples were wrapped and refrigerated for 24 h, after which they were allowed to reach room temperature. Shear force was measured using the Meullenet-Owens Razor Shear (MORS) blade method (29) with a texture analyser (TA-XT2, Texture Technologies Corp., Hamilton, MA, USA) using the cooked sample with an original dimension (raw samples) of 7.5 × 4.5 × 4.5 cm. Each sample was sheared at six locations on the sample surface (perpendicular to the muscle fiber direction) and then averaged.

### Statistical Analyses

Linear mixed models were used to analyze the effect of post mortem activity on turkey meat quality. The models for the nine meat quality traits (pH_initial_, pH_ultimate_, pH_delta_, L^*^, a^*^, b^*^, drip loss, cooking loss, and shear force) included the fixed effects of activity (scores 1–4), genetic line (A–F), and season (summer or autumn). To prevent overfitting of the model and provide insight into meat quality differences between different seasons, the slaughter date (*N* = 28) of the birds was categorized into seasons. Slaughter dates in June, July, and August were categorized as “Summer” (*N* = 3,897) and dates in September, October, and November (*N* = 1,545) were categorized as “Autumn.” Interactions between fixed effects were not considered due to the limited data available for possible two-way and three-way interactions.

All traits were modeled using a Gaussian distribution, except for drip loss and cooking loss for which a lognormal distribution and the back-transformed least-square means (LSmeans) presented. The α level for determination of significance was 0.05, and tendencies are reported between 0.05 and 0.10. All analyses were performed using SAS (Studio, version 9.4., SAS Institute Inc., Cary, NC, USA).

## Results

### Post Mortem Activity Scores

For intra-observer reliability, the Spearman correlation between the activity scores for the two sessions was 0.83. The simple and weighted kappa values were 0.80 ± 0.039 and 0.81 ± 0.039, respectively, indicating good reliability. The activity of most birds across all seasons and genetic lines was scored as 1 (86.9%, none to mild, Table 2). Scores 2, 3, and 4 made up 3.8, 7.2, and 2.0% of the birds, respectively.

**Table 2:** Number of birds (*N*_Birds_) with each post-mortem activity score (1, none to minimal; 2, mild; 3, moderate; 4, severe) for male turkeys from six genetic lines (A–F).

	**Activity**				
**Line**	**1**	**2**	**3**	**4**	**Total**
A	431 (82.1%)	19 (3.6%)	64 (12.2%)	11 (2.1%)	525
B	993 (86.6%)	43 (3.7%)	81 (7.1%)	30 (2.6%)	1,147
C	781 (88.4%)	48 (5.4%)	35 (4.0%)	19 (2.6%)	883
D	673 (86.8%)	38 (4.9%)	51 (6.6%)	13 (1.7%)	775
E	1,196 (88.0%)	36 (2.6%)	96 (7.1%)	31 (2.3%)	1,359
F	656 (87.2%)	23 (3.1%)	67 (8.9%)	6 (0.8%)	752
Total	4,730 (86.9%)	207 (3.8%)	394 (7.2%)	110 (2.0%)	5,441

*Numbers in brackets refer to percent of birds per score within each genetic line*.

### Effect of Post Mortem Activity on Meat Quality Traits

There was an effect of post mortem activity on a^*^, drip loss, and shear force (*P* < 0.05), however no effect of post mortem activity was detected for the remaining color measures (L^*^ and b^*^), pH, and physiochemical meat quality traits (*P* > 0.05, Table 3).

**Table 3:** Linear models for the effect of post mortem activity (score 1–4), genetic line (A–F), and season (summer vs. autumn) on turkey meat quality traits (*N*_Birds_= 817–895 depending on trait).

	***F***	***Num df***	***Den df***	***P***
a*				
Activity	3.61	3	876	0.0130
Line	6.43	5		<0.0001
Season	60.61	1		<0.0001
**Drip loss (%)**				
Activity	3.38	3	884	0.0178
Line	12.70	5		<0.0001
Season	10.75	1		0.0011
**Shear force (** ***N*** **)**				
Activity	3.19	3	881	0.0231
Line	0.29	5		0.9193
Season	4.83	1		0.0283
**pH**_**initial**_				
Activity	2.00	3	852	0.1757
Line	30.62	5		<0.0001
Season	7.59	1		0.0028
**pH**_**ultimate**_				
Activity	0.61	3	878	0.6077
Line	29.96	5		<0.0001
Season	20.56	1		<0.0001
**pH**_**delta**_				
Activity	1.35	3	807	0.2565
Line	19.28	5		<0.0001
Season	2.22	1		0.1366
L*				
Activity	1.64	3	870	0.1783
Line	2.11	5		0.0619
Season	75.96	1		<0.0001
b*				
Activity	0.47	3	873	0.7025
Line	12.96	5		<0.0001
Season	6.78	1		0.0094
**Cooking loss (%)**				
Activity	0.53	3	873	0.6589
Line	2.22	5		0.0508
Season	10.32	1		0.0014

*F statistic (F), numerator degrees of freedom; (Num df), denominator degrees of freedom (Den df, same for all factors within a model), and P-value, (P) are presented for each trait model*.

Initial pH (pH_initial_) was recorded at 45 min post mortem. All other traits were recorded at 24 h post mortem. Color traits (L*, a*, b*) were measured using D50 illumination.

The studied pH traits (pH_initial_, pH_ultimate_, and pH_delta_) were unaffected by post mortem activity (*P* > 0.05). The pH_initial_ of the meat from score 4 birds was lower than the other scores (e.g., −0.15, score 1 vs. score 4), however this was not significantly different as shown in Table 4.

**Table 4:** Differences in meat quality attributes between turkeys (*N*_Birds_= 817–895 depending on the trait) with different post mortem activity scores 1–4.

**Trait^1^**	**Activity**			
	**1**	**2**	**3**	**4**
**pH^2^**				
*N*_Birds_	782	44	56	98
pH_initial_	6.33 ± 0.02^A^	6.32 ± 0.04^A^	6.34 ± 0.04^A^	6.18 ± 0.07^A^
pH_ultimate_	5.73 ± 0.01^A^	5.73 ± 0.02^A^	5.75 ± 0.02^A^	5.72 ± 0.03^A^
pH_delta_	0.61 ± 0.03^A^	0.60 ± 0.05^A^	0.60 ± 0.04^A^	0.46 ± 0.08^A^
**Color^3^**				
*N*_Birds_	773	44	56	13
L*	37.66 ± 0.23^A^	37.28 ± 0.40^A^	37.08 ± 0.37^A^	37.99 ± 0.65^A^
a*	0.43 ± 0.05^A^	0.59 ± 0.09^A^	0.55 ± 0.09^A^	0.72 ± 0.15^A^
b*	1.67 ± 0.08^A^	1.71 ± 0.14^A^	1.56 ± 0.13^A^	1.73 ± 0.22^A^
**Physiochemical**				
*N*_Birds_	783	44	55	13
Drip loss (%)	1.13 ± 0.11^A^	1.16 ± 0.19^A^	1.46 ± 0.23^A^	2.09 ± 0.55^A^
Cooking loss (%)	29.76 ± 0.44^A^	29.75 ± 0.75^A^	30.47 ± 0.71^A^	29.88 ± 1.19^A^
Shear force (*N*)	9.43 ± 0.28^B^	8.43 ± 0.61^AB^	8.81 ± 0.45^AB^	9.13 ± 1.04^AB^

*Data is presented as LSmean ± SEM*.

1 *Within a given trait, line LSmeans that do not share a letter superscript are significantly different (P < 0.05)*.

2 *pH_initial_ was measured at 45 min post mortem and pH_ultimate_ was measured at 24 h post mortem. pH_delta_ is the difference between the two measurements*.

3 Color traits (L*, a*, and b*) measured at 24 h post mortem using D50 illumination.

Of the color traits (L^*^, a^*^, b^*^), a^*^ was affected by post mortem activity. While the results from the ANOVA for the linear model for a^*^ indicated that there was an effect of activity (*F*_3, 876_ = 3.61, *P* = 0.0130, Table 3), the adjusted *P*-values for the *ad-hoc* pairwise comparisons between the scores were not significant (*P*>0.05). Birds with an activity score >1 (none-minimal activity, Table 1) had lower a^*^ values compared to the other scores (Table 4). As the activity score increased, the a^*^ value increased by 32% (score 2), 26% (score 3), 51% (score 4) from score 1.

Within the physiochemical traits, both drip loss and shear force were affected by post mortem activity (*P* < 0.05). Shear force was affected by activity (*F*_3, 881_ = 3.19, *P* = 0.0231). Birds with score 1 produced meat with higher shear force compared to score 2 (*t*_3, 881_ = 2.63, *P* = 0.0425) but all other comparisons were not different (*P* > 0.05). Shear force was ~11% higher in meat from score 1 birds compared to score 2 (Table 4). For drip loss, the adjusted *P*-values for the comparisons indicated that the scores were not significantly different from one another (*P* > 0.05). However, birds with activity score 4 tended to have higher drip loss (~1%) than birds with score 1 (*t*_884_ = −2.49, *P* = 0.0618, Table 4).

### Effect of Genetic Line on Meat Quality Traits

There was an effect (*P* < 0.05) of genetic line on all the meat quality traits except for L^*^, cooking loss, and shear force (Table 3). Genetic line influenced all the studied pH traits (Table 5). Lines E and F demonstrated higher pH_initial_ compared to all other lines (*P* < 0.05), except for line B. Line A had lower pH_ultimate_ compared to lines C, E, and F. The largest difference in pH_ultimate_ was observed between line A and line F (+0.14, *t*_5, 878_ = −3.32, *P* = 0.0122). Line E also demonstrated a higher pH_ultimate_ compared to line C (+0.04, *t*_5, 878_ = −4.97, *P* < 0.0001). For the difference between pH_initial_ and pH_ultimate_ (pH_delta_), line E showed a greater pH_delta_ compared to line A (+0.15, *t*_5, 807_ = −5.45, *P* < 0.0001), line C (+0.19, *t*_5, 807_ = −9.26, *P* < 0.0001), and line D (+0.32, *t*_5, 807_ = −3.30, *P* = 0.0129). There was also a tendency for birds from line F to have a greater pH_delta_ compared to line D (+0.37, *t*_5, 807_ = −2.64, *P* = 0.0883).

**Table 5:** Differences in meat quality attributes between six genetic lines (A–F) of turkeys (*N*_Birds_= 817–895 depending on trait).

**Trait^1^**	**Line**					
	**A**	**B**	**C**	**D**	**E**	**F**
***pH^2^***						
*N*_Birds_	177	19	323	12	379	21
pH_initial_	6.20 ± 0.03^A^	6.24 ± 0.07^AB^	6.22 ± 0.02^A^	6.21 ± 0.07^A^	6.42 ± 0.02^B^	6.47 ± 0.06^B^
pH_ultimate_	5.66 ± 0.01^A^	5.73 ± 0.03^ABC^	5.73 ± 0.01^B^	5.74 ± 0.04^ABC^	5.77 ± 0.01^C^	5.80 ± 0.04^BC^
pH_delta_	0.55 ± 0.03^A^	0.61 ± 0.08^AB^	0.49 ± 0.03^A^	0.36 ± 0.10^A^	0.68 ± 0.03^B^	0.73 ± 0.11^AB^
**Color^3^**						
*N*_Birds_	171	19	307	7	376	6
L*	38.19 ± 0.24^A^	37.89 ± 0.54^A^	38.04 ± 0.22^A^	35.78 ± 0.85^A^	37.86 ± 0.21^A^	37.25 ± 0.92^A^
a*	0.64 ± 0.06^A^	0.62 ± 0.13^AB^	0.56 ± 0.05^A^	0.67 ± 0.20^AB^	0.41 ± 0.05^B^	0.53 ± 0.21^AB^
b*	2.10 ± 0.08^A^	1.98 ± 0.19^ABC^	1.55 ± 0.07^B^	1.13 ± 0.29^BC^	1.72 ± 0.07^B^	1.53 ± 0.32^ABC^
**Physiochemical**						
*N*_Birds_	176	19	311	5	380	6
Drip loss (%)	1.95 ± 0.19^A^	2.67 ± 0.58^A^	1.08 ± 0.09^B^	1.02 ± 0.41^ABC^	1.45 ± 0.12^C^	0.96 ± 0.35^ABC^
Cooking loss (%)	30.22 ± 0.44^A^	30.77 ± 1.04^A^	29.56 ± 0.39^A^	28.86 ± 1.76^A^	30.53 ± 0.39^A^	29.90 ± 1.67^A^
Shear force (*N*)	9.31 ± 0.28^A^	8.89 ± 0.61^A^	9.18 ± 0.45^A^	8.33 ± 1.04^A^	9.20 ± 0.24^A^	8.78 ± 1.00^A^

*Data is presented as LSmean ± SEM*.

1 *Within a given trait, line LSmeans that do not share a letter superscript are significantly different (P < 0.05)*.

2 *pH_initial_ was measured at 45 min post mortem and pH_ultimate_ was measured at 24 h post mortem. pH_delta_ is the difference between the two measurements*.

3 Color traits (L*, a*, and b*) measured at 24 h post mortem using D50 illumination.

There was a tendency for L^*^ to be influenced by genetic line at the factor level (*F*_5, 870_ = 1.64, *P* = 0.0619), although the adjusted *P*-values from the pairwise comparisons revealed no differences between the lines at *P* > 0.05 (Table 5). For a^*^, line E had lower a^*^ values than lines A (*t*_5, 876_ = 5.02, *P* < 0.0001) and C (*t*_5, 876_ = 4.02, *P* = 0.0009). There were also some differences in b^*^ between the lines. Line A had a higher b^*^ value than lines C (+0.55, *t*_5, 873_ = 7.60, *P* < 0.0001), D (+0.97, *t*_5, 873_ = 3.34, *P* = 0.0114), and E (+0.38, *t*_5, 873_ = 5.41, *P* < 0.0001). Additionally, line C had a lower b^*^ value than line E (−0.17, *t*_5, 873_ = −2.73, *P* = 0.0406), although the magnitude of the difference was not as large.

There was an effect of genetic line on the amount of drip loss (*F*_5, 884_ = 12.70, *P* < 0.0001), but not cooking loss (*F*_5, 873_ = 2.22, *P* = 0.0508) or shear force (*F*_5, 881_ = 0.29, *P* = 0.9193, Table 5). Lines A and B had the highest mean amount of drip loss (1.95 ± 0.188% and 2.67 ± 0.583%, respectively). Line A had greater drip loss than line C (+0.87%, *t*_5, 884_ = 7.04, *P* < 0.0001) and line E (+0.50%, *t*_5, 884_ = 3.67, *P* = 0.0035). Line B also had greater drip loss than line C (+1.59%, *t*_5, 884_ = 4.33, *P* = 0.0002) and line E (+1.22%, *t*_5, 884_ = 2.94, *P* = 0.0390). Lastly, line C had lower drip loss compared to line E (−0.37%, *t*_5, 884_ = −4.33, *P* = 0.0002). Although line F had numerically the lowest drip loss, there was no statistical difference between the other lines (*P* > 0.05), possibly due to the relatively large standard error. The mean cooking loss (30.0%) was consistent between the six lines with a standard deviation of 0.70%. The same was also observed for the mean shear force (9.0 N) of the lines which had a standard deviation of 0.36 N.

### Effect of Season on Meat Quality Traits

Season (summer vs. autumn) affected all the studied meat quality traits except for pH_delta_ (Table 3). In general, meat pH (pH_initial_ and pH_ultimate_) was higher during the summer season (Table 6). Although for both pH_initial_ and pH_ultimate_, the magnitude of the difference between the LSmeans is small (pH_initial_: +0.05, *t*_1, 861_ = −3.00, *P* = 0.0028; pH_ultimate_: +0.03, *t*_1, 861_ = −4.53, *P* < 0.0001). In the summer, meat had greater mean L^*^ (+1.39, *t*_1, 870_ = −8.72, *P* < 0.001), a^*^ (+0.29, *t*_1, 876_ = −7.79, *P* < 0.001), and b^*^ (+0.06, *t*_1, 873_ = −2.60, *P* = 0.0123) values compared to autumn. Drip loss, cooking loss, and shear force all increased during the summer (*P* < 0.05, Table 6). The mean drip loss and cooking loss were higher by 0.30% (*t*_1, 884_ = −3.28, *P* = 0.0011) and 0.93% (*t*_1, 873_ = −3.21, *P* = 0.0014) in the summer, respectively (Table 6). The shear force for the summer (LSmean: 9.14 ± 0.347 N) was greater than in autumn (LSmean: 8.75 ± 0.355 *N*, *t*_1, 881_ = −2.20, *P* = 0.0283, Table 6).

**Table 6:** Differences in meat quality attributes in male turkeys (*N*_Birds_= 817–895 depending on trait) between the summer (June–August) and autumn (September–November) seasons.

**Trait^1^**	**Season**			
	**Summer**	**Autumn**
**pH^2^**		
*N*_Birds_	604	299
pH_initial_	6.32 ± 0.04^A^	6.27 ± 0.03^B^
pH_ultimate_	5.75 ± 0.01^A^	5.72 ± 0.02^B^
pH_delta_	0.58 ± 0.04^A^	0.55 ± 0.04^A^
**Color^3^**		
*N*_Birds_	600	286
L*	38.20 ± 0.29^A^	36.81 ± 0.31^B^
a*	0.72 ± 0.07^A^	0.43 ± 0.07^B^
b*	1.74 ± 0.10^A^	1.60 ± 0.11^B^
**Physiochemical**		
*N*_Birds_	603	290
Drip loss (%)	1.57 ± 0.19^A^	1.27 ± 0.17^B^
Cooking loss (%)	30.43 ± 0.57^A^	29.50 ± 0.59^B^
Shear force (*N*)	9.14 ± 0.35^A^	8.75 ± 0.36^B^

*Data is presented as LSmean ± SEM*.

1 *Within a given trait, line LSmeans that do not share a letter superscript are significantly different (P < 0.05)*.

2 *pH_initial_ was measured at 45 min post mortem, and pH_ultimate_ was measured at 24 h post mortem. pH_delta_ is the difference between the two measurements*.

3 Color traits (L*, a*, and b*) measured at 24 h post mortem using D50 illumination.

## Discussion

The goal of this study was to examine the prevalence of post mortem activity in turkeys at a commercial processing plant and analyze the relationship between activity and meat quality, while accounting for differences between genetic lines (A–F) and seasons (summer vs. autumn). Post mortem activity was scored on a 1–4 scale adapted from McNeal et al. (5) after evaluating intra-observer reliability. Most birds displayed none/minimal post mortem activity (score 1, 87%) with lesser percentages having mild (score 2, 4%), moderate (score 3, 7%), and severe (score 4, 2%) muscle activity.

### Post Mortem Activity and Meat Quality

Meat redness (a^*^), drip loss (%), and shear force (N) were associated with activity score. Birds exhibiting severe flapping behavior had breast meat that was numerically redder than the other scores. A connection between meat redness and wing flapping was reported in broiler (5, 8, 30) and turkey studies (24, 25). While it has yet to be fully determined why vigorous wing flapping tends to increase breast meat redness, it is possible that these movements cause damage to the breast muscle as it is the main muscle responsible for wing movement. Reduced muscle tension during slaughter reduces blood spots, hemorrhaging and small blood vessels rupturing in the muscle so it is logical to assume that vigorous movement during bleeding increases muscle tension and subsequently redness (31). Similar to what was noted by McNeal et al. (5), although the differences between these values are numerically significant, they may not be of practical significance or able to be discerned visually by the average consumer.

Breast meat drip loss increased as activity score increased with the highest average drip loss being from birds that exhibited severe body movement and flapping (score 4). Vigorous muscle movement is known to deplete glycogen stores and more quickly increase muscle lactate which speeds up the rate of post mortem pH decline (14, 32, 33). This effect was documented by Berri et al. (30) who found that initial meat pH was strongly and negatively correlated with the duration of wing-flapping before slaughter. In turkeys, rapid declines in muscle pH are well-documented to decrease water-holding capacity of meat and result in greater drip loss and cooking loss (34–36). To provide further support for this hypothesis, Landes et al. (37), found that turkeys that were anesthetized with phenobarbital before slaughter (no struggle) had a slower rate of post mortem pH decline and subsequently lower shear force values. We were unable to discern an effect of post mortem activity on any of the studied pH traits. However, the average pH_initial_ for birds with activity score 4 was the lowest of all the scores. The lack of discernable effect may be due to the low number of birds who displayed vigorous post mortem activity. It is also possible that the ante mortem activity has a more substantial effect on meat pH than post mortem activity. Future studies should assess both ante and post mortem activity on turkey meat quality to determine their relative importance and relationship. It is unclear whether turkeys exhibiting post mortem activity are also more likely to be active before slaughter.

The toughness of cooked poultry meat (measured by shear force) has been demonstrated to increase with activity during slaughter (24). In our study, we found that birds with no/minimal activity (score 1) had higher average shear force compared to birds with mild activity (score 2), however this difference was small (1 N). Average shear force then increased between scores 2, 3, and 4 as post mortem activity increased, although these differences were not statistically significant. Other turkey studies have found that pre-slaughter activity increases the shear force of breast meat and the opposite is found if birds are anesthetized before slaughter to eliminate struggling (24, 25). This is likely due to the close relationship of shear force with pH and water-holding capacity. Rapid drops in initial pH reduces water-holding capacity which can result in a tougher meat product (higher shear force) (38).

Overall, the results of the present study indicate that post mortem activity may influence some meat quality traits. Although, the magnitude of these differences are not large, their cumulative effect on breast meat quality may be more substantial. Future work should consider how various stunning methods or settings influence the degree of post mortem activity in turkeys.

### Effect of Genetic Line

Genetic line influenced pH_initial_, pH_ultimate_, pH_delta_, a^*^, b^*^, and drip loss. There was no effect of genetic line observed for L^*^, cooking loss, and shear force. The genetic lines used in this study are purebred lines selected for different breeding goals. The most relevant difference for meat quality is the difference in average body weight at slaughter between the lines. The largest lines are sire lines F (25.2 ± 1.8 kg) and E (24.1 ± 1.6 kg), followed by dam lines B (21.9 ± 1.4 kg) and A (21.1 ± 1.4 kg). The two smallest lines are line C (18.8 ± 1.2 kg) and line D (14.6 ± 1.0 kg).

Differences in meat quality between genetic lines has been demonstrated in broiler chickens (12, 39), but consistent differences are not reported in literature for turkeys (21, 40–42). A study looking at fast- and slow-growing turkey lines was unable to detect any effect of genetic line on breast meat color (L^*^, a^*^, b^*^), drip loss, and shear force (40). Zampiga et al. (42) compared a variety of meat quality traits between two commercial turkey hybrids and found that the majority of traits (pH_ultimate_, L^*^, a^*^, marinade uptake, cooking loss, and shear force) were not influenced by genotype. However, some traits such as b^*^ and drip loss were different between the groups. Another study by Updike et al. (41) found no differences in the cooking loss, however shear force was significantly lower in the random bred control line compared to modern lines selected for bodyweight and breast yield. Similarly, we did not observe any difference in cooking loss between the six lines. Although we found that the heaviest lines (E and F; 8.8–9.2 N) had numerically higher shear force compared to the lightest line (line D; 8.3 N) these differences were not statistically significant. This could be because line D may be the lightest line in terms of weight, however it is still a modern turkey line with specific breeding goals, whereas Updike et al. (41) they were using a line which had been randomly bred and is more representative of a 1960's commercial turkey line. However, a different study of fast- and slow-growing turkey lines found that slow-growing turkeys had similar pH measurements as the fast-growing line, but were paler and had greater drip loss (21). In our study, the smallest and slowest growing line (line D) had a lower pH_initial_ compared to the fastest growing lines (lines E and F). Additionally, we found that the slowest growing line (line D), demonstrated the lowest average lightness and highest average redness numerically compared to the faster growing lines. This follows an opposite trend to paler meat observed in slow-growing turkeys by Fernandez et al. (21). However, there were no other differences between line D and lines E and F, which is in line with the lack of differences between fast- and slow-growing turkeys observed in Werner et al. (40).

Poultry meat quality may be influenced by body size and growth rate due to differences in fiber size, although there seems to be substantial conflict in the literature as to whether fast growth rate negatively impacts turkey meat quality (40). Faster growing chicken strains have a larger fiber cross-sectional area (43–46). In broiler chickens, genetic lines with greater breast muscle mass (larger fiber area) revealed lower glycogen stores and subsequently lighter colored meat that was less red and had higher ultimate pH (39). Given the differences in the literature, it may be helpful for future studies to incorporate a serial slaughter experimental design with different genetic lines to better assess how these meat quality attributes change over the different growth trajectories in turkeys. It should also be acknowledged that more data was available for lines A, C, and E compared to lines B, D, and F which may make it a challenge to draw conclusions about meat quality differences between certain genetic lines. Due to the observational nature of the study, and its collaborative nature as part of a larger project (47), the collection of meat quality data was concentrated on some lines more than others.

### Effect of Season

Season has a well-documented relationship with poultry meat quality and exposure to high ambient temperature before slaughter tends to produce meat that is lighter with decreased water-holding capacity (15, 48). We expected that turkeys slaughtered during the summer season would be exposed to higher temperature, especially during transportation, which may influence their meat quality. Indeed, season influenced all the studied meat quality traits (except for pH_delta_) in the current study, albeit to a limited extent.

Breast meat from birds slaughtered during summer has been reported to be lighter in color, less red, with a lower pH, and greater moisture loss (17, 18). Some of these results were shown in our study as we found the average L^*^ to be greater during summer, as well as a higher b^*^, indicating that meat is paler and more yellow in color. These effects have been demonstrated in another study of Canadian turkeys where the authors found that the mean L^*^ measurement was highest during the summer, lowest in the winter, with intermediate values in the spring and autumn (48). This aligns with increased incidence of pale, soft, and exudative (PSE) meat reported during summer compared to cooler seasons (18, 48). In Brazil, de Carvalho et al. (18) reported a 22% increase in the occurrence of PSE meat from turkeys processed during the summer compared to winter; i.e., which corresponded to a higher L^*^ and lower pH reported in the summer. To provide further support, turkey hens exposed to cold (−18°C) had breast meat that was darker (lower L^*^) compared to birds exposed to control temperatures (49). Interestingly, no effect of cold exposure on meat color was observed for turkey toms in this study which indicates that there may be an influence of sex on susceptibility to temperature stress.

Furthermore, we also report that juice loss (drip loss and cooking loss) and shear force were greater during summer months. It is believed that these effects on color and water-holding capacity occur because heat stress increases the conversion of glycogen to lactic acid in the muscle which causes rapid declines in muscle pH (6, 16). The fast decline in pH denatures the muscle proteins and pigments and results in a paler color and increased moisture loss in the raw and cooked products (34, 35).Conversely, meat that is characterized as dark, firm, dry (DFD), which is more often reported during the colder seasons (higher muscle glycogen use to keep the birds warm during transport), tends to have a higher pH and consequently is darker, redder, with decreased moisture loss (50).

However, contrary to expectations, meat pH was higher during the summer compared to autumn. Although the magnitude of this difference was very small (+0.03–0.05 units). As discussed above, we expected that higher summer temperatures would result in lower pH because of the faster conversion of glycogen to lactic acid (51), but also because cold exposure limits muscle glycogen which reduces the potential lactic acid formation in the meat (35). Turkey breasts classified as “fast glycolytic” (pH_initial_ < 6.2) have been reported to have a warmer internal temperature compared to “slow glycolytic” breasts and one can possibly expect that warmer muscles would be more likely in the summer season (51). It is possible that the pH results from our study do not align with other reports (17, 18) because we are comparing summer and autumn, which may be more similar in environmental conditions compared to the Canadian summer and winter. For example, the average temperature near the processing plant in 2019 for the summer months was 25.2°C compared to 12.9°C in autumn and −0.9°C in winter. This effect was somewhat demonstrated by McCurdy et al. (48) who found that the largest difference in L^*^ values for turkey breast meat samples was between the summer and winter seasons whereas L^*^ values in the spring and autumn were intermediate. Moreover, the majority of data in this study was collected during the summer due to the observational nature of the study and collaboration with the abattoir as part of a larger project (47). Future experimental (i.e., non-observational) studies with a more balanced data distribution may be beneficial to gain further insight into seasonal meat quality differences in Canadian turkeys.

It is also possible that some of the observed differences in meat quality are due to differences in growth between the seasons. After accounting for the effect of genetic line, the LSmean slaughter weight in autumn was 138 grams heavier than the summer and the LSmean breast weight was approximately 88 grams heavier in autumn (data not shown). Cooler temperatures are typically beneficial for poultry growth because the risk of heat stress, which has an inhibitory effect on feed intake and growth, is much lower (52). As mentioned previously, differences in growth and growth rate have the potential to affect meat quality. In particular, faster-growing broiler chicken strains have been shown to have lighter meat with higher ultimate pH (39). It is possible that the differences in growth between the two seasons may influence the effect on meat quality.

There are many points throughout the turkey production cycle where season may influence growth and meat quality. This is a factor of critical importance in many turkey-producing areas that experience distinct seasonal changes. While it should be acknowledged that the observed effects of season on the meat quality traits were small, they may be important when considering the cumulative impact of seasonal changes on turkey production. In the summer season, heat stress can be more likely due to higher ambient temperatures. Heat stress can negatively impact feed intake and growth (52) and result in health and wellbeing issues as well as death during transport (53). The occurrence of dead-on-arrivals at the processing plant was shown to be 79% higher for turkeys slaughtered during the summer compared to autumn (0.52 vs. 0.29%) (54). Therefore, even though the effects on most meat quality traits in this study were relatively small, combined with the effects of temperature on growth and mortality these impacts may be more significant to turkey producers than currently estimated.

## Conclusion

This study provides an initial assessment of the prevalence of post mortem activity during conventional turkey slaughter and the effect of this activity on meat quality traits. It is important to acknowledge that although we found associations between activity and several meat quality traits (a^*^, drip loss, and shear force), the magnitude of the effects was not necessarily large; however, their cumulative effect on profitability of a large turkey operation could be significant. Furthermore, the prevalence of birds exhibiting post mortem activity (score > 1) was low which may have influenced the results. In any case, this study indicates that vigorous post mortem activity may negatively impact certain meat quality traits in male turkeys. There was also a discernable effect of genetic line and season on most studied traits which identifies several areas of future research into the effects of different growth trajectories during different seasons on turkey meat quality.

## Data Availability Statement

The raw data supporting the conclusions of this article will be made available by the authors, without undue reservation.

## Ethics Statement

The animal study was reviewed and approved by the University of Guelph Animal Care Committee (AUP 3782).

## Author Contributions

CB, BW, SB, and EL conceived and designed the experiment. CB and BW secured funding for the experiment. EL, RV, NS, and JM conducted the experiment. EL analyzed the data and wrote the original draft. CB, BW, SB, NS, RV, and EL reviewed and approved the final manuscript.

## Funding

This project was funded by the Government of Canada through Genome Canada and the Ontario Genomics Institute (OGI-133). This study was part of the project entitled “Application of genomic selection in turkeys for health, welfare, efficiency, and production traits” funded by the Government of Canada through the Genome Canada Genomic Application Partnership Program and administered by Ontario Genomics [Recipients: BW (Industry) and CB (Academic)].

## Conflict of Interest

JM and BW were employees of Hybrid Turkeys at the time of the study. The funders had no role in the design of the study; in the collection, analyses, or interpretation of data; in the writing of the manuscript, or in the decision to publish the results. The remaining authors declare that the research was conducted in the absence of any commercial or financial relationships that could be construed as a potential conflict of interest.

## Publisher's Note

All claims expressed in this article are solely those of the authors and do not necessarily represent those of their affiliated organizations, or those of the publisher, the editors and the reviewers. Any product that may be evaluated in this article, or claim that may be made by its manufacturer, is not guaranteed or endorsed by the publisher.
